# Indigenous Data Governance: Strategies from United States Native Nations

**DOI:** 10.5334/dsj-2019-031

**Published:** 2019

**Authors:** Stephanie Russo Carroll, Desi Rodriguez-Lonebear, Andrew Martinez

**Affiliations:** 1 Native Nations Institute at the Udall Center for Studies in Public Policy, University of Arizona, US; 2 Mel and Enid Zuckerman College of Public Health, University of Arizona, US; 3 College of Social and Behavioral Sciences School of Sociology, University of Arizona, US

**Keywords:** Indigenous Data Sovereignty, Indigenous Data Governance, Sovereignty, Indigenous Sovereignty, Data Sovereignty, Data Stewardship

## Abstract

Data have become the new global currency, and a powerful force in making decisions and wielding power. As the world engages with open data, big data reuse, and data linkage, what do data-driven futures look like for communities plagued by data inequities? Indigenous data stakeholders and non-Indigenous allies have explored this question over the last three years in a series of meetings through the Research Data Alliance (RDA). Drawing on RDA and other gatherings, and a systematic scan of literature and practice, we consider possible answers to this question in the context of Indigenous peoples vis-á-vis two emerging concepts: Indigenous data sovereignty and Indigenous data governance. Specifically, we focus on the data challenges facing Native nations and the intersection of data, tribal sovereignty, and power. Indigenous data sovereignty is the right of each Native nation to govern the collection, ownership, and application of the tribe’s data. Native nations exercise Indigenous data sovereignty through the interrelated processes of Indigenous data governance and decolonizing data. This paper explores the implications of *Indigenous data sovereignty and Indigenous data governance* for Native nations and others. We argue for the repositioning of authority over Indigenous data back to Indigenous peoples. At the same time, we recognize that there are significant obstacles to rebuilding effective Indigenous data systems and the process will require resources, time, and partnerships among Native nations, other governments, and data agents.

## Introduction

Data is synonymous with life in a modern society, including official national statistics, surveys, social media posts, lab results, shopping preferences, voter registrations, school enrollment, IP addresses, and the list continues. These data have become the new global currency, and a powerful force in making decisions and wielding power (IEAG 2014, The Economist 2017). As the world moves toward open data, big data reuse, and data linkage, what do data-driven futures look like for communities historically plagued by data inequities? This paper draws on strategies from Native nations in the United States and combines expertise honed through meetings at Research Data Alliance plenaries and elsewhere to answer this question in four main ways. First, we define key terms and explore the evolution of Indigenous peoples’ rights to Indigenous data sovereignty. Second, we extend the Indigenous data sovereignty conversation to Indigenous data governance. Third, we describe the relationship between decolonizing data, Indigenous data governance, and rebuilding Native nations. Fourth, we provide case studies of Indigenous data governance occurring at tribal and non-tribal entities. Finally, we conclude with recommendations to strengthen Indigenous data governance for both Indigenous and non-Indigenous entities.

## Defining Key Terms

### Indigenous Peoples

It is first necessary to describe the vastly diverse Indigenous peoples and communities to which we refer in this paper. While there are many definitions of Indigenous peoples, including those that have been imposed by non-Indigenous entities, one of the more universally accepted “working definitions” is stipulated in the United Nations’ Martinez Cobo Study:

“Indigenous communities, peoples and nations are those which, having a historical continuity with pre-invasion and pre-colonial societies that developed on their territories, consider themselves distinct from other sectors of the societies now prevailing on those territories, or parts of them. They form at present non-dominant sectors of society and are determined to preserve, develop and transmit to future generations their ancestral territories, and their ethnic identity, as the basis of their continued existence as peoples, in accordance with their own cultural patterns, social institutions and legal system” (Martinez Cobo 1982).

Worldwide, there are over 370 million Indigenous peoples in over 90 countries representing more than 5,000 distinct cultures (United Nations 2009). This paper focuses specifically on the United States, where 5.2 million individuals self-identified as American Indian or Alaska Native (AIAN) alone or in combination with other races in the 2010 US Census (Norris et al. 2012). Individuals that reported AIAN heritage alone, known as the single race AIAN population, comprised 2.9 million people (49% of those that reported any AIAN heritage). In 2018, the United States government recognized 573 Native nations, 342 tribes in the lower 48 states with the remaining in Alaska (Federal Register 2019). A further 60 tribes have been recognized by state governments (National Conference of State Legislatures 2016). Many more Native nations and communities remain unrecognized by the settler colonial state, including those in the state of Hawai’i.

### Indigenous Data

Data are not a foreign concept in the Indigenous world. Indigenous peoples “have always been data creators, data users, and data stewards. Data were and are embedded in Indigenous instructional practices and cultural principles” (NCAI 2018, p. 1). For example, many Indigenous knowledge systems were based on generations of data gathering through observation and experience that then informed Indigenous practices, protocols, and ways of interacting with other people and with the natural world. The translation of knowledge into data was similarly evident. Indigenous data were recorded in oral histories, stories, winter counts, calendar sticks, totem poles, and other instruments that stored information for the benefit of the entire community (Rodriguez-Lonebear 2016).

Indigenous data systems *center on interdependence*, not the acquisition of individual knowledge. While the acquisition and transmission of knowledge by individuals is necessary to support the collective base, Indigenous data systems rely on shared responsibilities to ensure that Indigenous ways of knowing, being, and doing are transmitted from one generation to the next. Within this context, knowledge belongs to the collective and is fundamental to who Indigenous nations are as peoples. Similarly, data that inform Indigenous ways of knowing are also collectively held. While individuals hold knowledge (stories, songs, knowledge of special relationships with the natural world), they have roles and responsibilities to the collective to steward this knowledge (Cajete 2000).

We consider Indigenous data to be *“any facts, knowledge, or information about a Native nation and its tribal citizens, lands, resources, cultures, and communities. Information ranging from demographic profiles, to educational attainment rates, maps of sacred lands, songs, and social media activities,”* (Rainie et al. 2017b, p. 1) among others. Indigenous data comprise information and knowledge about our environments, tribal citizens and community members, and our cultures, communities, and interests (Nickerson 2017). The definition encompasses both collective and individual level data. It also highlights that the concrete boundaries between data, information, and knowledge as defined in Western contexts are more fluid in an Indigenous context; Indigenous data extend far beyond bits and bytes (De Beer 2016) and have implications for the governance of both data born digital and that which emerges from knowledge, lanugae, and information.

### Data Dependency

The legacy of colonialism remains a central organizing force in Indigenous-settler relations across the world. In the United States, this legacy is evident in the demarcation of reservation lands, long-standing economic inequality, vast environmental violations, and persistent Indigenous health disparities, among numerous other measures (Deloria and Lytle 1983). A less explored area is the role of data as a tool to marginalize Indigenous peoples, eradicate their ways of life, and rewrite their histories to advance the colonial project (Bruhn 2014). Throughout the Indigenous world, epistemicide, or the killing and co-optation of knowledge systems, has occurred and continues today (Sousa Santos 2007). Examples in the United States include, federal policies of assimilation, forced removal, relocation, residential schooling and other cultural ruptures that sought to erase Indigenous knowledge and forced many tribes to rely on external sources of information about their communities’ economic, environmental, and health status. The resultant state of data dependency for US Native nations is precipitated by such processes of colonization.

Data dependency is sustained through a paradox of scarcity and abundance: extensive data are collected about Indigenous peoples and nations, but rarely by or for Indigenous peoples’ and nations’ purposes (Walter 2018, United Nations 2018). Many of these data do not recognize or privilege Indigenous worldviews, or benefit Indigenous peoples (United Nations 2018). As a result, Indigenous data ecosystems are characterized by:

- Inconsistent, inaccurate, and irrelevant data for Indigenous peoples;
- External control and ownership of data;
- Community mistrust of data resulting from exploitative research and policies;
- Lack of external support for data infrastructure and capability; and,
- Data that describe Indigenous peoples and lifeways through a deficit lens (Rodriguez-Lonebear 2016, Rainie et al. 2017c, Kukutai and Taylor 2016, Walter 2016).

The suppression, usurpation, and co-optation of Indigenous knowledge systems perpetuates the data divide, furthering data dependency and maintaining the paradox of scarcity and abundance. Indigenous data sovereignty disrupts the current paradigm, offering a way forward to shift power dynamics and realize Indigenous goals and visions.

### Indigenous Data Sovereignty

In mainstream usage, “data sovereignty is the concept that information which has been converted and stored in binary digital form is subject to the laws of the country in which it is located” (Rouse 2013). This definition focuses on geographic jurisdiction over digitized data. A nation-state’s laws control the digital data that is housed within its geographic boundaries. Indigenous data sovereignty extends beyond this mainstream definition. It is not limited by geographic jurisdiction or digital form.

*“Indigenous data sovereignty is the right of Indigenous peoples and tribes to govern the collection, ownership, and application of their own data”* (Rainie et al. 2017b). Further, it refers to all data gathered by the Native nation themselves or by other external data agents. “Indigenous data sovereignty (IDS) derives from the inherent right of Native nations to govern their peoples, lands, and resources” (NCAI 2018, p. 1). Some settler-colonial governments, particularly those in English settler societies, recognize the right of Indigenous peoples to govern via treaties and other legal mechanisms negotiated on a nation-to-nation basis with Native nations. The concept of IDS as a collective right also is positioned within an international Indigenous rights framework. IDS “accords with international declarations and covenants to which the United States has become a signatory, such as the United Nations Declaration on the Rights of Indigenous Peoples (UNDRIP),” (NCAI 2018, p. 2) specifically Articles 3, 4, 5, 15(i), 18, 19, 20(i), 23, 31, 32, 33, 38, and 42 (Davis 2016).

While the inherent right to exercise IDS extends to all Native nations in principle, the extent to which Indigenous peoples can exercise IDS is constrained by their position in the problematic settler colonial paradigm of recognition and acknowledgment. Therefore, IDS is a site of tension and opportunity that signals a departure from the mainstream construct of government recognition towards inherent sovereignty and self-determination.

The genesis of Indigenous data sovereignty lies in the traditions, roles, and responsibilities for the use of collectively held information (Kukutai and Taylor 2016). IDS aligns with the concept of peoplehood, which positions Indigenous peoples within a complex system of inter-relationships among sacred histories, ceremonial cycles, ancestral homelands, and continuously evolving cultural traditions and languages (Cajete 2000, Holm et al. 2003). Peoplehood also underscores Indigenous nations’ inherent responsibilities to the system of inter-relationships, to the natural world, and to their peoples. Through this communal lens, Indigenous peoples conceptualize IDS not only as a right, but also as a responsibility.

Unlike racial or ethnic groups, Indigenous peoples and nations are political entities with rights and interests in data about their peoples, lands, and resources (Banerjee 2003, United Nations 2018, Rainie et al. 2019). The status as political entities is the fundamental difference in the relationship between Indigenous peoples and nations with Indigenous data, and other racial and ethnic groups’ relationships with data about their populations and peoples. As IDS asserts Indigenous nations as rightsholders of their data, it challenges dominant data discourses, articulating power and colonial dynamics within data agendas that apply outside Indigenous contexts (Rainie et al. 2019).

## Indigenous Data Governance

Now, we explore Native nation governance and its relationship to IDS. Governance of data is an extension of the rights and practices that originate in the sovereignty of nation states. As sovereigns, governance of data in an Indigenous context is within the purview of tribal governments. However, positivist approaches to knowledge acquisition and dissemination often sustain and support the use of mainstream policy, research, and evaluation. Such privileging of Western science and other mainstream knowledge systems favors current power dynamics, maintaining the paradox of data scarcity and abundance and perpetuating data dependency. IDS deliberately repositions control of data back to Indigenous peoples. It is at the center of a growing global movement created, nurtured, and designed to be Indigenous led (Kukutai and Taylor 2016). IDS challenges the power dynamic inherent in Western data systems that continue to disenfranchise Native knowledge systems and Indigenous people, providing space for the data governance that reflects Indigenous nations’ voices, values, and vision (United Nations 2018). Thus, overcoming data dependency requires acknowledging tribal sovereignty and supporting Indigenous data governance, and making changes across the data ecosystem to data processes, ownership, access, and control.

Through a western lens, governance can be defined as “the system of values, policies and institutions by which a society manages its economic, political and social affairs through interactions within and among the state, civil society and private sector. It is the way a society organizes itself to make and implement decisions” (United Nations Development Programme 2011). Governments are the structures that carry out these functions, which include tribal governments. In the United States, tribes are sovereign nations. Each tribe has its own government that makes and enforces laws and policies. Tribal governance structures vary from theocracies to chiefdoms to tripartite systems akin to Western governance models (Duthu 2008). We acknowledge that Indigenous governance systems vary across global contexts. However, the destructive impacts of colonization on governance infrastructure and leadership mechanisms are universal. As such, there is value beyond US context in understanding the intersection of governance and data sovereignty.

Indigenous governance systems have been undergoing processes of reclamation of self-rule and increased self-determination over the last fifty years. This movement has been referred to as Native nation rebuilding (Jorgensen 2007). It occurs as tribes ‘enhance their foundational capacity’ to make and implement strategic decisions about their own affairs. It is a comprehensive effort to rebuild Indigenous societies that work on Indigenous nations’ terms in the continued wake of colonization. This includes political, economic, social, and cultural development that requires accurate and relevant tribal data (Rodriguez-Lonebear 2016, Kukutai and Taylor 2016, Rainie et al. 2017c, United Nations 2018). We argue that the core of a nation’s ‘foundational capacity’ must include strong tribal data systems.

**Figure 1:**
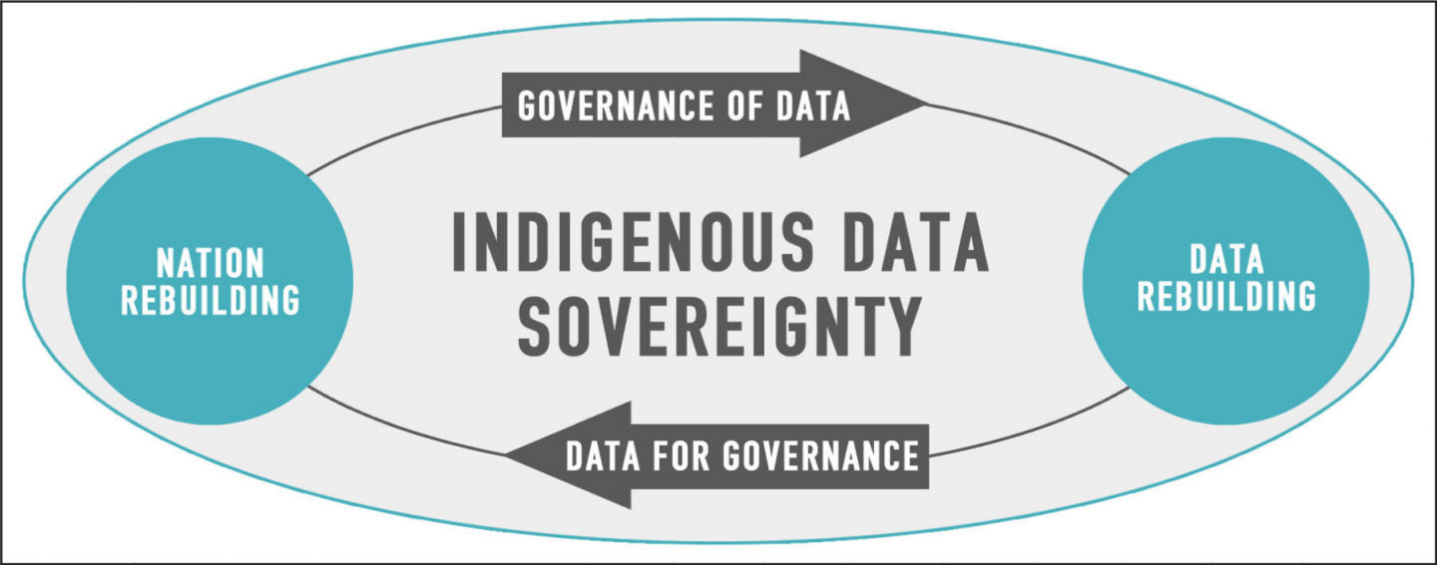
Reclaiming Indigenous Data Sovereignty Through Indigenous Data Governance and Decolonizing Data.

Indigenous data sovereignty is an aspiration to be achieved through data governance (see Figure 1). Native nations exercise IDS through the interrelated processes of Indigenous data governance and decolonizing data. Decolonizing data occurs as Indigenous nations and other data agents replace external, non-Indigenous norms and priorities with Indigenous systems that define data, and inform how it is collected and used. It results in findings–derived both from Indigenous data collected externally and from internal data produced by Indigenous nations–that reflect the understandings of those peoples. Indigenous data governance is the act of harnessing tribal cultures, values, principles, and mechanisms–Indigenous ways of knowing and doing–and applying them to the management and control of an Indigenous nation’s data ecosystem (Rainie et al. 2017b, Walter et al. 2018). “Indigenous data governance is decision making. It is the power to decide how and when Indigenous data are gathered, analysed, accessed and used” (Walter et al. 2018, p. 3).

The processes of decolonizing data and reclaiming Indigenous data governance seen in Figure 1 are not linear nor are they purely parallel. As technology advances, data are decolonized, new data are created, and changes in tribal government activities occur, the process will continue to evolve and the need for new Indigenous mechanisms of data governance that honor and protect data arise. This process of decolonizing data and the mechanisms of Indigenous data governance will be continuously revisited, revised, and remembered (in the case of traditional cultural methods of data governance). The extent to which Indigenous nations are engaging in these processes varies as we describe later in the paper.

### Data Governance and Nation Rebuilding

**Figure 2:**
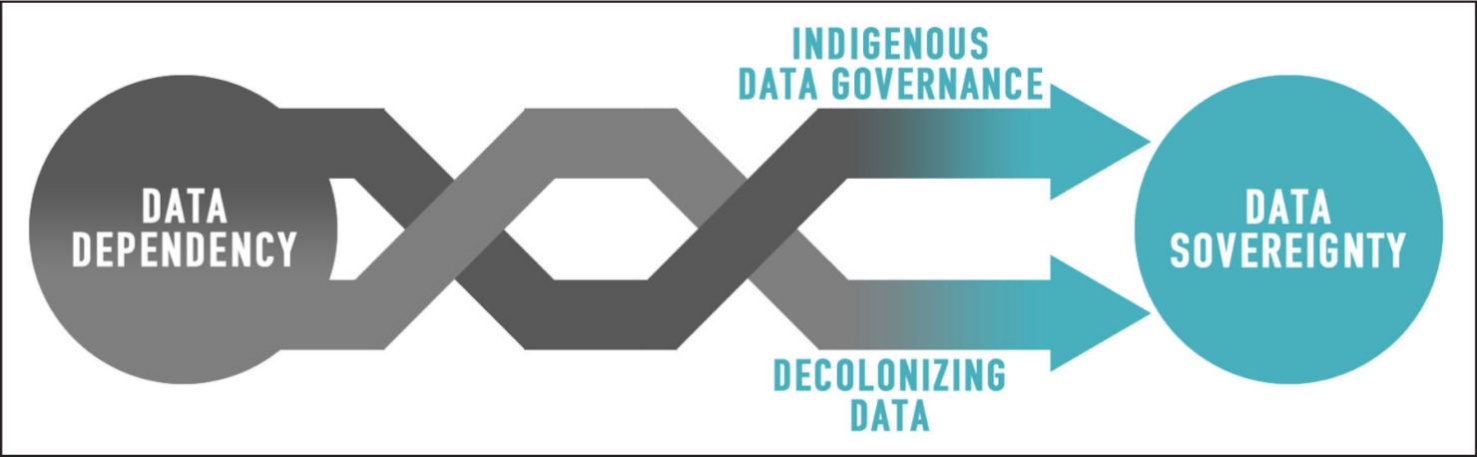
Data and Governance: The Interdependence of Nation Rebuilding and Data Rebuilding.

Like other nation states, tribal governments carry out the multitude of tasks that comprise governance (Cornell et al. 2004). Key among these tasks is making decisions about one’s citizens, communities, and resources; doing so requires data. Indigenous data sovereignty epitomizes the connections among Indigenous data, data governance, and Native nation rebuilding (see Figure 2) (Rainie et al. 2017b). Tribes need accurate, relevant, and timely data for policy and decision-making; that is, data for governance (Smith 2016). Tribes also need mechanisms to honor, protect, and control their information both internally and externally; that is, governance of data. As tribes rebuild their governance institutions, they increase their capability to govern their data, which in turn, facilitates stronger evidence-based decision-making. Indigenous data governance can thus be described as a reciprocal relationship between data for governance and governance of data. The first is a matter of quality, relevance, and access: can Native nations obtain the data they need for governance? The second is a matter of ownership and control: can Native nations manage, protect, and use that data? Tribal sovereignty and IDS sit at the center of this relationship.

### Data for Governance

Data for governance is a central need and, therefore, investment of most nation states. In the United States, for example, the decennial census is arguably the premier source of data for governance among federal, state, and local governments. The US Census is the largest peacetime mobilization that the government undertakes (Thompson 2015). The data generated from this $13 billion operation are used to, among other things, calculate political apportionment, disperse billions of dollars in federal funding, and plan the nation’s economic and social development portfolios.

Data for governance raises the question: what data do nations need to govern effectively? Often, the data to which tribes have access are not relevant nor accurate for their governance needs (Rodriguez-Lonebear 2016, Rainie et al. 2017c). For example, the US Census notoriously undercounts American Indian and Alaska Native peoples, particularly those living on tribal lands (Lujan 2014). American Indians and Alaska Native peoples experienced the highest undercount (4.9%) of any racial or ethnic population in the 2010 Census (US Census 2012). Further, the US Census captures self-identification, which is not the same thing as tribal citizenship. As nations and polities, tribes have defined citizenship criteria. Not all people who self-identify as American Indian or Alaska Native are citizens of a tribe. Thus, the value of the US Census as an accurate and relevant count of tribal populations for Native nation governance is questionable.

A population census is not the only source of data for governance. Administrative data collected through routine government interactions, including voting, obtaining a driver’s license, paying taxes, and accessing federal services are another powerful source for governance, as are survey and other research data. However, equal access to these data and linkage with existing data held by Native nations are not guaranteed. Native nations do not have ready access to the data collected by external agents about their citizens, lands, and resources (Rodriguez-Lonebear 2016, Rainie et al. 2017c, Walter 2016). Disparate access is compounded by the limited capability of many Native nations to build their own data systems that support governance. The underlying power structures enabling or inhibiting data development, data legitimization, and data access leads us to the importance of governance of data.

### Governance of Data

The mainstream discussion of data governance, to date, has largely encompassed corporate data governance, where data governance serves corporate goals of efficiency and profit (Khatri and Brown 2010). Public entities have reconfigured corporate data governance to reflect their values–policy and service improvements, cost reduction, and ensuring and easing compliance (Bruhn 2014). Data governance in the Indigenous world, however, serves a different set of goals. IDG is about the people–arguably a nation’s most valuable resource–and purpose. In addition to governing data with the intent to use the data for decision-making, Indigenous peoples’ steward data in order to protect their cultural knowledge and sustain their way of life for past, present, and future generations (Smith 2016). Therefore, instead of aligning with corporate values, IDG aligns with Indigenous communities’ core values; reciprocity and stewardship are higher priority than efficiency and economic growth.

Within Indigenous data governance efforts, the control of data plays a critical role in exercising IDS. Opportunities exist along a spectrum of data governance models in which tribes can exert increasing levels of control: 1) zero to low control in data commons scenarios, such as open data sets required by federal funders; 2) equal control in partnerships; and 3) full control over proprietary data, such as tribal enrollment records (Bruhn 2014).

While tribes may ultimately have little control over data generated by large corporations who wield data hierarchies (e.g., Facebook, Amazon), they can strive to exert control in scenarios where no or low control might have been presumed. For instance, the National Institutes of Health (2014) requires all genomic data generated by NIH funded research projects to be deidentified and submitted for inclusion in a secondary data set. However, if tribal laws or policies require a tribe to retain ownership of all data generated by research projects, then tribes can request to have those data exempted from broad data sharing, including submission to federal secondary databases. This is a clear example how tribal control of data via Indigenous data governance mechanisms–tribal laws and policies regarding data–can influence the way others (e.g., researchers and the federal government) interact with tribal data.

Open data, big data, and broad data sharing all create challenges to Indigenous nations’ control of their data ecosystems. These data trends and others sit at the nexus of issues around colonization, bias, and lack of knowledge about Indigenous rights. Data communities often view these challenges in binaries: data are open or not, data are useful or not, and data are included in data sets or not. For Indigenous peoples, the risks and benefits exist at both ends of the binary. If Indigenous data are not included in large datasets, such as genomics metadata, then Indigenous peoples will be invisible and may not realize the benefits of emerging health technologies and advances. On the other hand, if data about Indigenous peoples in big data and open data are used without guiding rights and interests frameworks, Indigenous peoples risk representation that reflects the bias in existing data ecosystems. IDS as operationalized through IDG provides a framework and mechanisms for protecting Indigenous rights with respect to data and promoting ethical use of data for development according to Indigenous values and interests. Solutions require engagement with Indigenous peoples and the use of IDG principles by others when stewarding Indigenous data (Smith 2016, Rainie et al. 2019, Garrison et al. 2019).

Tribal data governance faces challenges other than control. Indigenous nations encounter data capability, capacity, and funding shortfalls, resulting in limited resources for governing data. Such resource constraints complicate tribal decisions about data governance, including what data to control, whether to exert authority over that data, how to manage and protect the data, and how to use it. However, evaluating the likelihood of gaining control over the data, the sensitivity of the data, and the accessibility of data can assist tribes in deciding when to utilize limited resources in order to govern data (Hudson et al. 2018). For example, the Māori Te Mana o te Raraunga Framework from Aotearoa/New Zealand provides a guide for determining data governance methods and levels of control/accessibility in order to ensure trusted use of secondary data (Hudson et al. 2018). Data determined as highly sensitive via this framework require active approaches to data governance through Māori control of data or partnerships with Māori to control data; moderately sensitive data might demand less active data governance and looser control such as consultation with or notification of Māori when data are used. Further, Māori may choose low or no control data governance mechanisms such as data commons or open data for data that are deemed not sensitive.

Relationships are also at the core of IDG (Smith 2016). Indigenous peoples and nations are more than mere stakeholders (Banerjee 2003); as sovereign polities, they are *rightsholders* with the right to govern data about their peoples, lands, and resources, choosing what when, how, and how much control to exert. That right is the fundamental difference in the relationship between Indigenous peoples and non-Indigenous stakeholders with regard to Indigenous data (Banerjee 2003, Rainie et al. 2019).

IDG has implications for how tribes internally govern their data and their influence over how others steward data about tribes, their peoples, lands, and resources. Exercising the right to IDS and implementing effective IDG occurs within a larger data ecosystem in which other governments, corporations, and entities also control tribes’ data. Many Indigenous and non-Indigenous organizations steward Indigenous nations’ data. Indigenous stakeholders include Indigenous-led or serving organizations and IDS networks. For example, Indigenous organizations such as urban Indigenous organizations and other Native-serving nonprofits collect and store data about their participants. Non-Indigenous stakeholders include nation-states and other governments, researchers, and NGOs. Stakeholders have interests in Indigenous data and at times govern or steward Indigenous data, but they do not have the inherent sovereign right to govern and choose when and how to control that data. Tribes establishing and maintaining relationships with these stakeholders is a critical aspect of IDG. IDS also necessitates these stakeholders to incorporate tribal principles into their own data governance practices.

## Strategies for Indigenous Data Governance

In the continuous pursuit of Indigenous data sovereignty, Native nations are actively moving away from data dependency–the state of depending on other entities to provide data about the tribe and its peoples, communities, and resources (see Figure 1). Existing research has demonstrated that positive outcomes are evident in the cases where repositioning Indigenous control over data, knowledge, and information has occurred (Rainie et al. 2017c, Rodriguez-Lonebear 2016, Berry 2009, Champagne 2012, Cornell and Kalt 2007, Cross et al. 2004, Edwards et al. 2009, Galloway 1995, Harvard Project on American Indian Economic Development 2006, Krepps and Caves 1994, Wakeling et al. 2001). Strategies to reclaim and decolonize tribal data systems include, rebuilding community trust in research, improving data accuracy and quality, promoting Indigenous methodologies and epistemologies, developing local capability, supporting self-determination, and producing meaningful and relevant data for decision making (Rainie et al. 2017c). Next, we share results of a systematic search of Indigenous data governance strategies and efforts that Native Nations and others in the US are undertaking as they exercise IDS and decolonize data.

### Tribal Case Studies

Within the last five years, there has been increased awareness of IDS and a growing number of tribes exercising IDG (Rodriguez-Lonebear 2016, Rainie et al. 2017c, NCAIPRC 2017, NCAI 2018). The recent interest may be associated with growth in funding for data intensive projects, particularly in the age of big data and open data. So too could it correspond with an increased demand for better data as voiced by tribal leaders, Indigenous researchers, and community stakeholders (Rodriguez-Lonebear 2016, Rainie et al. 2017c). Tribes have also sought data that align with their values and vision, such as empowering families and improving health and wellbeing. To facilitate these pursuits, many tribes have either developed or adapted data governance mechanisms. In this section, we present the efforts of several tribes that engaged in tribal data governance. We also examine specific mechanisms that influence the governance and stewardship of Indigenous data by Native nations and non-Indigenous entities.

#### National Congress of American Indians Tribal Data Capacity Project

In 2014, the National Congress of American Indians Policy Research Center coordinated a pilot project to build tribal data capacity with five tribes across the country (NCAIPRC, 2017). Each tribe undertook a unique data project that supported their governance efforts. These projects included tribal population censuses, a “data gap” needs assessment, and a tribal version of the Behavioral Risk Factor Surveillance System household survey. The key recommendations from these five tribal data projects, as reported by the National Congress of American Indians, primarily target the federal government: more federal investment to support tribal data collection, analysis, and management; tribal authority to integrate federal program funds for comprehensive and streamlined data collection and management efforts; partnerships between federal agencies and tribes to achieve shared data aims; and intertribal forums to encourage the exchange of tribal data best practices. In addition to these overall recommendations, the governance mechanisms that these tribal nations deployed to successfully complete their projects are critical to examine as tribal nations increasingly turn to each other for guidance to advance IDS.

For example, the Pueblo of Laguna was the only tribe of the five to plan and execute their own census or survey (as opposed to hiring external consultants) (NCAIPRC, 2017). The Pueblo engaged in a unique partnership agreement with the University of New Mexico to develop a proprietary software program used by the tribe’s census enumerators. This is an example of an effective data governance mechanism that secures external expertise to develop cutting edge technology on tribal terms with tribal money (i.e. grant money obtained by the tribe) for tribal purposes. Their software was effective in achieving a high census response rate across their reservation. Notably, the software remains the property of the tribe and can be used for future data collections.

#### Swinomish Tribe Climate Change Initiative

The Swinomish Tribe in Washington illustrates another case of tribal data governance through their Swinomish Climate Change Initiative (Swinomish Climate Change Initiative undated, Donatuto et al. 2014). Since 2007, after a Proclamation by the Swinomish Indian Senate, the tribe has been studying the effects of climate change on their reservation. In 2014, the tribe engaged in a pilot study to evaluate climate change impacts along their shorelines and on the health of their people. The tribe examined key community health concerns and projected impacts to habitat and shoreline archaeological sites. By testing existing indicators of community health, they identified which ones were most relevant to their communities and proposed alternatives to the ones that were less relevant. In this case, the Swinomish tribe engaged in a formal partnership agreement with another Native nation, as well as with non-tribal researchers. This agreement and the decision-making processes and protocols that followed are effective governance mechanisms because both Native nations agreed that they retain complete ownership over their respective data and that data would not be used in analyses or released without prior review and approval by that tribe’s leadership. The tribes also are named as co-authors in all related publications (as opposed to an individual tribal leader or staff member), which is another means by which tribes can exercise control over the dissemination of research findings on their terms in academic and scientific fora.

#### Tribal Codes and Review: Navajo Nation Human Research Review Board

Tribal legislation and tribal research bodies, such as tribal institutional review boards, are governance mechanisms that wield some measure of influence over governance of Indigenous data. They do so by controlling access to research about tribal peoples, resources, and cultures, as well as by controlling research taking place on tribal lands. An example of an effective tribal research code is the Navajo Nation Human Research Review Board (NNHRRB). The Board has functioned to regulate, monitor, and control research within the Navajo Nation since 1996 (Navajo Nation 2009). A core element of the NNHRRB’s 12-step approval process is extensive engagement with community partners. Further, all data must be turned over to the Navajo Nation at the conclusion of the project.

### Non-tribal Case Studies

While data governance by tribes for tribes is advancing across Indian Country, a less developed area of Indigenous data governance asks: how can Native nations influence *external* governance and stewardship of Indigenous data? Answering this question requires discussion at the intersection of IDG, western legal frameworks, and corporate systems of management.

#### Federal Law

One of the most compelling, albeit elusive, mechanisms is the law. Federal laws like the Native American Graves Protection and Repatriation Action of 1990 (NAGPRA) provide a strong legal mechanism requiring external institutions that receive federal funding to return possession and control of precious tribal data: cultural items, including human remains, funerary objects, and other sacred objects unlawfully obtained from Native American homelands. Penalties for non-compliance are steep–up to 12 months of prison time and a $100,000 fine. Since NAGPRA’s passage, nearly 60,000 individual human remains and over 1.7 million funerary objects have been repatriated to Indigenous communities (National Park Service 2018). By these accounts, NAGPRA is an effective data governance mechanism; yet NAGPRA took nearly twenty years to become law, and there have been few pieces of legislation since with the same sweeping mandate for compliance by non-Indigenous entities with respect to Indigenous data.

#### Guides and Guidelines

Other governance mechanisms are more administrative in nature, such as partnership agreements and the adoption of guiding principles and values, which are often catalyzed by an ethical impetus. For example, the School for Advanced Research developed a set of Museum and Community Collaboration Guidelines in partnership with Native and non-Native museum professionals, cultural leaders, and artists. The Guidelines offer principles for building successful collaborations between Indigenous communities and museums. Important considerations are addressed, such as following cultural protocols, being flexible, extending hospitality, ensuring appropriate compensation for expertise, and understanding that access to knowledge is not a universal right (The School for Advanced Research 2018). Similarly, the National Congress of American Indians (2012) developed a guide for doing research in tribal communities entitled, “Walk Softly, Listen Carefully: Building Research Relationships with Tribal Communities.” This guide can be considered a road map of diverse strategies for doing meaningful and responsible research with tribes. It includes a set of core values in conducting research with tribes: “Indigenous knowledge is valid and valued; culture is always a part of research and thus research cannot be culturally neutral; responsible stewardship includes the task of learning how to interpret and understand data and research; tribes must exercise sovereignty when conducting research and managing data; and research must benefit Native people” (NCAIPRC and MSUCNHP 2012, p. 10).

### Urban, Inter Tribal, and Supra-tribal Cases

Indigenous organizations also have a critical role in promoting IDS and IDG. Located in metropolitan areas, embedded in Indian Country, or in key state or federal government hubs, Indigenous entities such as inter tribal organizations, urban Indian entities, and supra-tribal groups are uniquely positioned to advocate for IDG and IDS in spaces where external entities are often located.

#### Urban Indian Health Institute

Urban organizations serve citizens of Native nations in diaspora (in addition to numerous other populations), and thus share a degree of common constituency with tribes. The Urban Indian Health Institute (UIHI) is an organization that actively partners with Native nations to decolonize data and improve health outcomes for American Indians and Alaska Natives living in metropolitan areas. UIHI’s Data Dashboard uses national surveillance data to visualize health disparities across a range of indicators (UIHI 2018). In addition to providing needed information on Urban Indians, the UIHI’s Data Dashboard is an example of a tool that Native Nations may wish to pursue to advance data governance at the tribal level.

#### Albuquerque Area Southwest Tribal Epidemiology Center

The Albuquerque Indian Health Board’s Albuquerque Area Southwest Tribal Epidemiology Center is an example of an entity decolonizing data and pursuing IDG partnerships between a non-profit and tribes. Guided by a board comprised of tribal leaders, the Center uses Indigenous quantitative methodologies aligned with IDS to develop Indigenous data collection instruments, support Native nations in improving data used to make funding decisions, and “serve as translators and intermediaries between community and non-Native partners” (Walter and Suina 2018).

#### National Congress of American Indians

Finally, in June 2018, the National Congress of American Indians, the largest tribal membership organization in the US, passed a resolution (KAN-18–011) in “Support of US Indigenous Data Sovereignty and Inclusion of Tribes in the Development of Tribal Data Governance Principles” (NCAI 2018). This is the first collective action and statement in the US to “support the efforts of tribes to exercise Indigenous data sovereignty and governance, the efforts to advocate for and provide research on Indigenous data sovereignty, and support the inclusion of tribes in the development of any broad principles of tribal data sovereignty and/or governance” (NCAI 2018, p. 1). The resolution sets the initial agenda for both pantribal and Native nation movement toward IDS via IDG.

#### International Indigenous Data Sovereignty Efforts

The international sharing of information and action around IDS is a critical pathway towards supporting IDG and decolonizing data. Such sharing requires a degree of goodwill and buy-in, especially for Indigenous peoples for whom the ‘sharing’ of data often looks much more like ‘taking” (Rodriguez-Lonebear 2016). The sharing of ideas, actions, and best practices for reclaiming and decolonizing data is occurring in the United States and elsewhere, particularly in Canada and Australasia (Rodriguez-Lonebear 2016; Rainie et al. 2017c, Kukutai and Taylor 2016, BCFNDGI 2018, FNIGC 2018, Nickerson 2018). However, it has not happened organically; rather, a persistent community of practice has been steering the course. The establishment of an Indigenous Data Sovereignty Interest Group through the Research Data Alliance (RDA IDS Group; rd-alliance.org) has provided a mechanism for in-person sharing, interaction with mainstream data users, and expansion to other global geographies, which has proven to be most effective in this context.

Currently three nation-state based Indigenous data sovereignty networks exist: Te Mana Raraunga, the Māori Data Sovereignty Network in Aotearoa/New Zealand (temanararaunga.maori.nz) formed in late 2015; co-authors Stephanie Russo Carroll and Desi Rodriguez-Lonebear launched the United States Indigenous Data Sovereignty Network (USIDSN; usindigenousdata.arizona.edu) in early 2016; and the Maiam nayri Wingara Aboriginal and Torres Strait Islander Data Sovereignty Collective in Australia (maiamnayriwingara. org) emerged in 2017. Similar initiatives are underway elsewhere, and the First Nations Information Governance Center in Canada has been a leading voice for the right of Indigenous peoples in relation to their data for over two decades (http://fnigc.ca/).

The USIDSN developed relationships with the RDA in 2016. Carroll convened an invited IDS workshop at the RDA Plenary at International Data Week, Denver, Colorado in 2016. The following year, Rodriguez-Lonebear was awarded an RDA Fellowship. Through the merging of connections leveraged through the USIDSN’s international relationships and with researchers at the RDA, the founders of the existing nation-state based networks joined to create the International Indigenous Data Sovereignty Interest Group at the Research Data Alliance (RDA IDS Group; rd-alliance.org). The group has convened at every RDA plenary since for a total of five RDA plenary sessions plus an International Data Week panel, which engaged over 100 participants. The RDA plenaries have facilitated the expansion of the IDS discussion beyond North America and Australasia to include Indigenous peoples in the Global South and Asia. In addition, the RDA IDS Group hosted a workshop at the RDA plenary at the International Data Week in Gaborne, Bostwana in late 2018. The workshop participants drafted broad, international principles for the governance of Indigenous data. The C.A.R.E. Principles of Indigenous Data Governance (collective benefit, authority to control, responsibility, ethics) are intended for adoption and implementation by international research and policy organizations in addition to and alongside the FAIR Principles (findable, accessible, interoperable, reusable) (Wilkinson et al. 2016).

## Recommendations to Strengthen Indigenous Data Governance

As these case studies demonstrate, Native nations are working toward Indigenous data sovereignty at various paces by implementing mechanisms of Indigenous data governance. In this process, tribes are developing principles of IDG, research protocols, research review boards, data sharing agreements, and data repositories. They also will continuously revisit, reuse, and revise these mechanisms, and others employed over millennia, to better achieve their goals. As the development of data governance principles and mechanisms expands, so too will opportunities for strengthening nation-to-nation relationships between Native nations and other governments, and between Indigenous and non-Indigenous stakeholders.

The following set of recommendations for the continued development of IDS and IDG is the product of two years of rightsholder and stakeholder engagement through the USIDSN and RDA. Rightsholders included tribes and Indigenous nations internationally. Stakeholders included Indigenous and non-Indigenous researchers, policy makers, data users, tribal leaders, and community activists. Specifically, we employed qualitative methods to analyze participant observation data from 27 gatherings in the United States and at RDA plenaries. Data included input on mechanisms for data governance, tribal data governance opportunities and challenges, and other stakeholders’ roles in bolstering tribal data governance efforts. Table 1 highlights recommendations. Tribal rightsholders must be involved in every recommended. To ensure equitable and beneficial outcomes, Indigenous peoples must have a decision-making role as policies, principles, and strategies are developed and when IDG mechanisms and infrastructure are established and implemented. Table 1 also illustrates how recommended actions are not the sole purview of Indigenous peoples. Non-Indigenous entities also have a role in some of these efforts.

Tribal rightsholders can decide their level of engagement with every recommendation based on cultural fit, vision, capacity, and resources. As tribal resources and capacity grow, so could the degree of engagement. There are also identified areas of overlap where involvement from both Indigenous and non-Indigenous stakeholders is beneficial. For example, every Indigenous nation has established cultural protocols around songs, stories, and ceremonies, regardless of whether those protocols have been codified into contemporary law. Those protocols should be communicated and honored by all parties. These recommendations are meant to serve as a starting point. It should be noted that this is not an exhaustive list, and it will continue to grow as rightsholders and stakeholders develop and implement their IDG frameworks.

This list of recommendations is nonlinear; it is not meant to be a step-by-step process. Every Native nation engages with these issues to some degree, and there is no wrong place to begin nor any wrong place to focus attention and resources. Some Native nations have identified specific areas to advance internally within the tribe, and they have deployed their resources accordingly. Other Native nations have chosen to partner with external Indigenous or non-Indigenous stakeholders to identify potential funding sources, develop their capacity, and build their IDG framework.

## Conclusion

Reclaiming Indigenous data sovereignty is a journey, not a destination. The journey will look different for each Native Nation as they identify their respective data needs and then determine how they can best meet those needs. Tribes will use many different mechanisms on their journey, just like one may take different modes of transportation, double back, and pick up various passengers along the way. Central to Indigenous data sovereignty, however, is that Native Nations always remain in the driver’s seat. Indigenous data governance and Indigenous data sovereignty are integral to rebuilding strong Native Nations. As tribes grow their data systems and expand their reach to external entities, financial support and technical expertise become even more critical to the mission. Without investment in people and infrastructure, Indigenous data governance is unlikely to be fully realized. This brings us back to the value of people and relationships. At its core, practicing Indigenous data governance is about being a good ancestor, partnering with other data stewards, and ensuring data-driven futures by Indigenous peoples for Indigenous peoples.

Operationalizing the recommendations requires investments by tribes, other governments, foundations, and other funders to support tribes in growing their own data capacity and capability, including hard infrastructure (e.g., servers) and soft infrastrucure (e.g., people and networks); codes, policies, and procedures; and strengthening relationships toward Indigenous led data governance. Next steps include the release of the C.A.R.E. Principles of Indigenous Data Governance, increased regional and issue-focused meetings to advance the Indigneous data governance agenda in the US, and the creation of US-based principles of Indigeous data governance.

Finally, and most importantly, approaches to enhance Indigenous data governance must recognize and promote sovereignty; lead with Indigenous core values; include dialogue comprised of multiple ways of knowing; utilize and support exiting tribal data governance protocols and procedures; engage and promote Indigenous scholarship; and conduct data science in service to communities. Fundamentally, enhancing Indigenous data governance efforts toward Indigenous data sovereignty require a commitment to changing the current power dynamics in open data, big data, data systems, and data science. We must make the invisible, visible by including and listening to Indigenous peoples and nations in data decisions and discussions that affect them, such as federal advisory committees, data science principles implementations (e.g., FAIR), and the creation of policies for open data, open science, and secondary data use.

## Figures and Tables

**Table 1:** Means of Advancing Indigenous Data Sovereignty and Governance via Responsible Actors.

**Tribal Rightsholders**

**Develop** tribe-specific data governance principles
**Develop** tribe-specific data governance policies and procedures
**Generate** resources for Indigenous data governance by tribes
**Stakeholders**

**Acknowledge** Indigenous data sovereignty as a global objective
**Build** an Indigenous data sovereignty framework that specifies the relationships among data processes such as collection, storage, and analysis.
**Create** intertribal institutions dedicated to data leadership and building data infrastructure and support for tribes
**Develop** mechanisms to facilitate effective Indigenous data governance
**Establish** data governance mechanisms that non-tribal governments, organizations, corporations, and researchers can use to support Indigenous data sovereignty
**Explore** the complexities of individual and collective rights in relation to Indigenous data sovereignty
**Explore** the relationships among ethics, law, data governance in relation to Indigenous data sovereignty
**Grow** financial investment in Indigenous data infrastructure and capability
**Identify** common principles of Indigenous data governance
**Incorporate** Indigenous data sovereignty rights into all rightsholders’ and stakeholders’ data policies
**Promote** adoption and implementation of common principles of Indigenous data governance by tribes, governments, organizations, corporations, and researchers within the United States
**Recruit and Invest** in data warriors (Indigenous professionals and community members who are skilled at creating, collecting, and managing data)
**Share** strategies, resources, and best practices
**Strengthen** domestic and international Indigenous data sovereignty and Indigenous data governance connections among Native nations and Indigenous peoples
